# Pain and depressive symptoms among adolescents: prevalence and associations with achievement pressure and coping in the Norwegian Ungdata study

**DOI:** 10.1186/s12889-024-20566-x

**Published:** 2024-11-05

**Authors:** Henriette Jahre, Margreth Grotle, Kaja Smedbråten, Kåre Rønn Richardsen, Britt Elin Øiestad

**Affiliations:** 1 Center for Intelligent Musculoskeletal Health, Department of Rehabilitation Science and Health Technology, Oslo Metropolitan University, Postboks 4 St. Olavs plass, Oslo, 0130 Norway; 2 Research and communication unit for musculoskeletal health (FORMI), Division of Clinical Neuroscience, Oslo University Hospital, Oslo, Norway

**Keywords:** Adolescent, Pain, Depression, Mental health, Pressure, Co-occurrence

## Abstract

**Background:**

This study investigated the prevalence of pain, depressive symptoms, and their co-occurrence in Norwegian adolescents. Additionally, we investigated if perceived achievement pressure and coping with pressure were associated with pain, depressive symptoms and co-occurrent pain and depressive symptoms.

**Methods:**

Cross-sectional data from the Norwegian Ungdata Survey (2017–2019) were analysed. Adolescents from across Norway completed an electronic questionnaire including questions on perceived achievement pressure, coping with pressure, pain, and depressive symptoms. Descriptive statistics presented prevalence rates, and multinominal regression reported in relative risk ratios (RR) was employed to estimate associations, adjusted for gender (boys/girls), school level, and socioeconomic status.

**Results:**

The analyses included 209,826 adolescents. The prevalence of pain was 33%, 3% for depressive symptoms, and 14% reported co-occurring pain and depressive symptoms. The prevalence of co-occurring symptoms was higher in girls (22%) than boys (6%). Significant associations were found between perceived achievement pressure and pain (RR 1.11, 95% CI 1.10–1.11), depressive symptoms (RR 1.27, 95% CI 1.27–1.28), and co-occurring symptoms (RR 1.34, 95% CI 1.33–1.34). Struggling to cope with pressure was associated with pain (RR 2.67 95% CI 2.53–2.81), depressive symptoms (RR 16.68, 95% CI 15.60-17.83), and co-occurring symptoms (RR 27.95, 95% CI 26.64–29.33).

**Conclusion:**

The prevalence of co-occurring pain and depressive symptoms is high among Norwegian adolescents. Perceived achievement pressure and struggling to cope with pressure were associated with isolated and, more strongly, co-occurring pain and depressive symptoms. Enhancing adolescents’ ability to cope with pressure could be a crucial target in treating pain and depressive symptoms.

**Supplementary Information:**

The online version contains supplementary material available at 10.1186/s12889-024-20566-x.

## Background

Pain and depressive symptoms are common in the adolescent population, with increasing prevalence rates in the last decades [1, 2]. Both conditions seem more prevalent among girls than boys and are more frequent in older adolescents [3]. These conditions pose tremendous consequences for the individuals and the society [2], and in many cases, co-occur. We previously found that 9.6% of Norwegian adolescents reported co-occurring neck/shoulder pain and depressive symptoms [3]. The high prevalence is worrisome, as studies have shown that individuals with these comorbidities often report additional problems such as higher disability, worse treatment outcomes, and lower return to work than individuals with single health complaints [4, 5]. Findings from a qualitative study of adolescents suggest that co-occurring pain and mental health problems are harder to manage and more socially isolating than isolated pain or mental health symptoms [6]. The reasons for the increase in prevalence of these conditions are unknown, but the high prevalence and consequences of co-occurring pain and depressive symptoms may indicate that this is a subgroup demanding special attention.

Adolescence is a vulnerable time of life where school, peers, and social media play important roles that influence how adolescents behave and feel about themselves [7, 8]. Factors related to adolescents’ social environment may be linked to adolescents’ health and potentially the co-occurrence of pain and depression. Several reports have highlighted that adolescents today are exposed to high pressure and demands of performing well in all areas, which may lead to a higher vulnerability to mental and physical complaints [9]. For example, studies have shown that especially girls report high pressure to do well at school, body appearance, and get “likes” on social media [10–12]. Still, the pressure itself do not necessarily lead to pain or poor health. Pressure can be used positively as a motivator, potential for approval, motivation to learn new skills and mastery experience [13]. However, if the pressure becomes too much to cope with, it can lead to a stress response, potentially leading to poor health [11, 13]. For example, studies have shown that too much school pressure and social pressure related to body appearance are associated with psychological distress, especially among adolescent girls [11].

To the best of our knowledge, the relationship between adolescents’ perception and coping with pressure and the co-occurrence of pain and depressive symptoms has not been previously investigated using large datasets. Most prior research has focused separately on either pain or depressive symptoms [14, 15]. To provide evidence-based practice for this patient group, we need more specific knowledge about the co-occurrence of pain and depressive symptoms. Describing this patient group is crucial for developing research hypotheses and establishing a foundation for future intervention studies.

The objectives of this study were to (i) investigate the prevalence of pain, depressive symptoms, and the co-occurrence of pain and depressive symptoms in Norwegian adolescents, stratified by gender and school level (ii) estimate the association between perceived achievement pressure and pain, depressive symptoms, and co-occurring pain and depressive symptoms, and (iii) estimate the association between coping with pressure and pain, depressive symptoms, and co-occurring pain and depressive symptoms.

## Methods

### Study design and setting

This study used cross-sectional data from the Norwegian Ungdata Survey, collected between 2017 and 2019 [16]. The Ungdata survey is a population-based study including adolescents from lower (13–15 years) and upper secondary school (15–19 years) from nearly all municipalities in Norway (95%). The survey is conducted by Norwegian Social Research (NOVA) and regional centers for drug rehabilitation (KoRus). The municipalities are encouraged to participate in the survey every third year, ensuring that each municipality only participated once during the period between 2017 and 2019. Data collection took place during school hours, administered by teachers, and involved a comprehensive electronic questionnaire covering topics such as parents, friends, school, local environment, lifestyle, leisure activities, health, and well-being [16]. The questionnaire consisted of a combination of previously validated questionnaires, and questionnaires developed specifically for the Ungdata survey [17]. This study’s reporting follows the Reporting of Observational Studies in Epidemiology (STROBE) guidelines [18].

### Study sample

All adolescents from lower and upper secondary school (13–19 years of age) in Norway were invited to participate in the survey. The response rate was 87% for lower secondary school and 73% for upper secondary school students [16]. The total number of Ungdata Survey responders was 251,047, but due to missing data on gender, pain, depressive symptoms, perceived pressure, or coping, 26,889 (10.7%) were excluded. Boys had more missing data on all study variables compared to girls (Additional file 1).

### Ethical considerations

Participation was voluntary. Parents of students younger than 16 years of age were informed about the study two weeks prior to data collection and could contact the school to opt their child out of participation. Data collected from lower secondary school students was anonymous, eliminating the need for approval from data protection agencies. Data collection for upper secondary school students received approval from the Norwegian Agency for Shared Services in Education and Research (SIKT). This approval was necessary because the survey collected detailed information regarding educational program, ethnicity, living situation, and parental work status. However, since the survey did not collect names or ages, written informed consent from the students was not required.

### Outcome

The outcomes were pain, depressive symptoms, and co-occurring pain and depressive symptoms.

Pain was measured by the item “*Have you had any of these health issues during the past month?”*, with the response categories “*headache*”, “*neck/shoulder pain*”, “*joint and muscle pain*”, and “*stomach ache*”. Each pain location had the responses “*never”*,* “a few times”*,* “many times”*, and *“daily”*. Responders answering having any of these complaints “many times” or “daily” were operationalized as “having pain”, and those who responded “never” or “a few times” were operationalized as “low pain”. Responders reporting one or more pain sites and having a score of depressive symptoms < 3.0 were operationalized as “pain”.

Depressive symptoms were measured using six items from the Depressive Mood Inventory Scale, derived from the Hopkins Symptom Checklist [19]: “*During the past week*,* have you been affected by any of the following issues: (i) felt that everything is a struggle*,* (ii) had sleep issues*,* (iii) felt unhappy*,* sad or depressed*,* (iv) felt hopelessness about the future*, (v) *felt stiff or tense*, (vi) *worried too much about things.”* Each of these items were answered on a Likert scale ranging from “*not at all* [1]” to “*very much* [4]”. The scores were summarised, and a mean score was calculated [1–4] and dichotomised into two levels with ≥ 3 as depressive symptoms. This operationalisation has been used in previous studies [3, 20, 21], and the scale is validated in adolescents [22]. Adolescents with a depression score of ≥ 3 and low pain were operationalized as “depressive symptoms”.

Co-occurring pain and depressive symptoms were included in a composite variable consisting of responses from the pain questionnaire and the Depressive Mood Inventory Scale. Respondents reporting one or more pain sites and scoring ≥ 3 on the Depressive Mood Inventory Scale were treated as cases with co-occurring pain and depressive symptoms in this study. The reference category consisted of adolescents reporting low pain and no depressive symptoms.

### Perceived achievement pressure

Perceived achievement pressure was measured by the question: “*Do you feel pressure in your everyday life?*” with four sub-categories: (i) *pressure to look good or have a good body*, (ii) *pressure to do well at school*, (iii) *pressure to do well at sports*, (iv) *pressure to have many followers and likes on social media*. The response alternatives were on a four-point scale: *No pressure (0*), *a little pressure* [1], *some pressure* [2], *a lot of pressure* [3], *very much pressure* [4]. These questions were summarized on a scale from 0 to 16 and used as a continuous variable in the analyses. This scale was developed specifically for the Ungdata survey and has demonstrated acceptable internal consistency (Cronbach’s alpha 0.75 boys, and 0.81 for girls) [23].

### Struggling to cope with pressure

Struggling to cope with pressure was measured by the question: “*Have you felt so much pressure during the past week that you have struggled to cope with it*?” The response alternatives were: *“never”*,* “sometimes”*,* “quite often”*, and *“very often”.*

The responses *“never”* and *“sometimes”* were merged and categorized as *“no”*,* and “quite often” and “very often”* were merged and categorized as *“yes”*, as suggested by the developers [23].

### Background variables and confounders

Gender was used as a dichotomous variable in the analyses (girls/boys) and socioeconomic status (SES) was measured using questions representing three different dimensions: both parents’ education, number of books at home, and level of prosperity. The scale goes from 0 to 3, where 0 means the lowest level of SES. This scale is used in other publications based on the Ungdata survey [24]. School level was a dichotomous variable categorized as lower or upper secondary school. Since Ungdata is an anonymous survey, data on age was not available.

### Statistical analyses

Descriptive analyses are presented with medians and interquartile ranges (IQR) for continuous variables and counts and percentages for categorical variables. The prevalence of pain, depressive symptoms, and co-occurring pain and depressive symptoms is presented as percentages. To investigate whether prevalence rates differed significantly between girls and boys, we conducted chi-squared tests. Multinominal regression analyses were conducted to investigate the associations between perceived achievement pressure, struggling to cope with pressure and pain, depressive symptoms, and co-occurring pain and depressive symptoms. Gender, SES, and school level were included as confounders in the adjusted analyses of the total sample. To assess potential gender differences in the associations, we tested for interactions between gender and achievement pressure, and gender and struggling to cope with pressure. Since there were significant interactions, analyses stratified by gender were conducted to further explore the actual differences and magnitudes of effects. SES and school level were included as confounders in the stratified analyses. We used relative risk ratios (RR) with 95% confidence intervals (CI) as measure of association, and *p*-values of < 0.05 were considered statistically significant. The analyses were conducted using STATA statistical software system, version 18 [25].

## Results

The total study sample consisted of 209,826 adolescents, 51,5% were girls, and 56,3% were from lower secondary school (Table 1). The median perceived achievement pressure score was 5.0 (IQR 6), and twice as high among girls (6.0, IQR 6) than boys (3.0, IQR 5). Responses to the sub-categories of pressure are reported in Additional file 2. Eleven per cent reported that they struggled to cope with the pressure, 16% of the girls and 5.5% of the boys (Table 1).

### Prevalence of pain and depressive symptoms

**Fig. 1:**
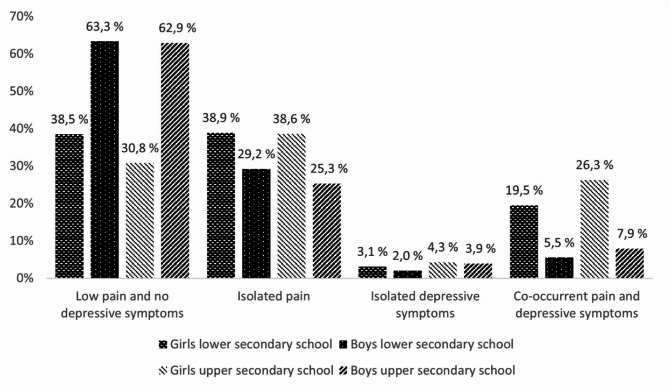
Prevalence of pain and depressive symptoms among Norwegian adolescents stratified by gender and school level

The overall prevalence of pain was 48%, and the overall prevalence of depressive symptoms was 18%. Thirty-three per cent reported isolated pain and three per cent reported isolated depressive symptoms. 14% reported co-occurring pain and depressive symptoms, 22 per cent of the girls, and 6 per cent of the boys. A statistically significantly higher prevalence of all conditions was observed in girls contrasted to boys (*p* < 0.001) (Fig. 1).

**Table 1:** Characteristics of the study participants (*n* = 209,826)

Variables	Girls	Boys	All
**Gender**,** n (%)**	108,071 (51.5)	101,755 (48.5)	209,826
**School level**,** n (%)**			
Lower secondary school	60,254 (55.8)	57,945 (56.9)	118,199 (56.3)
Upper secondary school	47,817 (44.2)	43,810 (43.1)	91,627 (43.7)
**Socioeconomic status (0–3)**	2.2 (0.7)	2.1 (0.7)	2.2 (0.7)
**Perceived pressure (0–16)** ^**α**^	6.0 (6)	3.0 (5)	5.0 (6)
**Struggle coping with pressure**,** n (%)**			
Yes	17,298 (16.0)	5602 (5.5)	22,892 (10.9)

Continuous variables are presented with median and interquartile ranges. ^α^Responses to each sub-category are presented in Additional file 2

### Perceived achievement pressure and pain and depressive symptoms

Statistically significant associations were found in both crude and adjusted analyses between perceived achievement pressure and pain, depressive symptoms and co-occurring pain and depressive symptoms (Table 2). Adjusted analyses revealed that for each unit increase in perceived achievement pressure the RR of pain was 1.11 (95%CI 1.10–1.11), 1.27 (95%CI 1.27–1.28) for depressive symptoms, and 1.34 (95%CI 1.33–1.34) for co-occurring pain and depressive symptoms compared to low pain and no depressive symptoms(Table 2).

The analysis revealed a significant interaction between gender and achievement pressure for depressive symptoms (*p* = 0.008), but not for pain (*p* = 0.189) or co-occurring pain and depressive symptoms (*p* = 0.230). Analyses stratified by gender revealed similar significant associations between perceived achievement pressure and pain, depressive symptoms, and co-occurring symptoms in both girls and boys (Table 2).

**Table 2:** Associations between perceived achievement pressure and pain and depressive symptoms

Outcome	All (*n* = 209,826)		Girls (*n* = 108,071)		Boys (*n* = 101,755)	
	Crude	Adjusted	Crude	Adjusted	Crude	Adjusted
	RR (95% CI)	RR (95% CI)	RR (95% CI)	RR (95% CI)	RR (95% CI)	RR (95% CI)
**Pain** ^a^	1.14 (1.13–1.14)	1.11 (1.10–1.11)	1.11 (1.10–1.11)	1.11 (1.11–1.11)	1.10 (1.10–1.11)	1.10 (1.10–1.11)
**Depressive symptoms** ^b^	1.29 (1.28–1.29)	1.27 (1.27–1.28)	1.27 (1.26–1.28)	1.28 (1.27–1.30)	1.26 (1.24–1.27)	1.26 (1.25–1.27)
**Co-occurrence**	1.39 (1.38–1.39)	1.34 (1.33–1.34)	1.33 (1.32–1.33)	1.34 (1.33–1.35)	1.33 (1.32–1.34)	1.33 (1.32–1.34)

^a^ isolated pain; ^b^Isolated depressive symptoms; Co-occurrence = co-occurring pain and depressive symptoms. Analyses for the total sample are adjusted for gender, socioeconomic status (SES) and school level. Analyses stratified for gender are adjusted for SES and school level. RR = Relative Risk Ratios, CI = Confidence intervals

### Struggling to cope with pressure and pain and depressive symptoms

The association between struggling to cope with pressure and pain, depressive symptoms, and co-occurring pain and depressive symptoms was statistically significant in both crude and adjusted analyses (Table 3).

Significant interaction effect of gender was found for depressive symptoms (*p* < 0.001) and co-occurrent pain and depressive symptoms (*p* < 0.001), but not for pain (*p* = 0.406). Analyses stratified by gender revealed RR of 2.68 (95%CI 2.47–2.91), 19.40 (95%CI 17.50-21.51), and 32.29 (95%CI 29.88–34.88) for pain, depressive symptoms, and co-occurring pain and depressive symptoms respectively among boys. Among girls the RR was 2.56 (95% CI 2.40–2.74) for pain, 14.84 (95%CI 13.60-16.19) for depressive symptoms, and 25.70 (95%CI 24.15–27.34) for co-occurring pain and depressive symptoms (Table 3).

**Table 3:** Associations between struggling to cope with pressure and pain and depressive symptoms

Outcome	All (*n* = 209,826)		Girls (*n* = 108,071)		Boys (*n* = 101,755)	
	Crude	Adjusted	Crude	Adjusted	Crude	Adjusted
	RR (95% CI)	RR (95% CI)	RR (95% CI)	RR (95% CI)	RR (95% CI)	RR (95% CI)
**Pain** ^a^	2.99 (2.84–3.14	2.67 (2.53–2.81)	2.56 (2.40–2.74)	2.56 (2.40–2.74)	2.68 (2.47–2.91)	2.68 (2.47–2.91)
**Depressive symptoms** ^b^	18.33 (17.16–19.57)	16.68 (15.60-17.83)	14.86 (13.62–16.21)	14.84 (13.60-16.19)	19.61 (17.70-21.72)	19.40 (17.50-21.51)
**Co-occurrence**	33.84 (32.31–35.45)	27.95 (26.64–29.33)	25.80 (24.25–27.44)	25.70 (24.15–27.34)	32.68 (30.25–35.30)	32.29 (29.88–34.88)

^a^ isolated pain; ^b^Isolated depressive symptoms; Co-occurrence = co-occurring pain and depressive symptoms. Analyses for the total sample are adjusted for gender, socioeconomic status (SES) and school level. Analyses stratified for gender are adjusted for SES and school level. RR = Relative Risk Ratios, CI = Confidence intervals

## Discussion

In this study, we found that 33% of Norwegian adolescents reported pain, 3% reported depressive symptoms, and 14% reported co-occurring pain and depressive symptoms. Co-occurring symptoms were more prevalent among girls than boys (22% versus 6%). There was a statistically significant association between perceived achievement pressure and pain, depressive symptoms and co-occurring pain and depressive symptoms, with the highest RR observed for co-occurring problems. The association was significantly stronger for depressive symptoms in girls compared to boys, but similar between genders for pain and co-occurring symptoms. Reporting struggle to cope with pressure was strongly associated with pain, depressive symptoms, and co-occurring symptoms, with the highest RR observed for adolescents with co-occurring problems. The association was significantly stronger among boys than girls for both depressive symptoms and co-occurring pain and depressive symptoms.

These findings show that one-third of adolescents reported pain without depressive symptoms, but only a minority of adolescents with depressive symptoms did not report pain, and co-occurring problems are common. The prevalence estimates for co-occurring pain and depressive symptoms correspond with other studies. A study from Sweden found that 50% of adolescents with depressive symptoms also experienced headache, 55% reported co-occurring abdominal pain, and 50% reported co-occurring back pain [26]. A population-based study of adolescents from China found a prevalence of co-occurring pain and depressive symptoms of 6.2%, somewhat lower than what we found in this study [27]. Reasons for these discrepancies may be due to different measurements, and definitions of pain.

Girls reported a higher prevalence of pain and depressive symptoms, and more than three times higher prevalence of co-occurring symptoms than boys. These gender differences are typically seen in studies of both pain and depressive symptoms [28]. Explanations for these differences might include combinations of affective, biological, and cognitive factors, such as emotional reactivity, pubertal timing and development, and cognitive style and rumination [29, 30]. Some also argue that girls in general have a lower threshold for reporting experiences as problematic, so findings could be influenced by differences in reporting style [29–31].

A comprehensive understanding of the mechanisms underlying the onset and progression of co-occurring pain and depression in adolescents remains limited. Soltani and colleagues proposed a conceptual model encompassing mutual factors that may contribute to the development and maintenance of co-occurrent pain and depression [28]. Their model underscores the bidirectional relationship between pain and depression, influenced by child-specific factors like neurobiological influences, cognitive, behavioral, and affective elements. Stress and adverse experiences are also highlighted in the model, acknowledging their impact on the experience and trajectory of both pain and depression. Crucially, this framework operates on the premise that these contextual and individual factors are interconnected in dynamic and intricate ways, likely interacting to influence and sustain both conditions [28]. Further, one recent twin study from Italy found that shared genetic and environmental factors best explained the co-occurrence of pain, anxiety, and depression in adolescents [32]. Some studies also highlight that the presence of one type of vulnerability may increase the likelihood of the development of another type of vulnerability, such as in co-occurring pain and depression [29]. Additionally, some studies find that depression in early life is a risk factor for developing pain conditions later in life [4].

There were significant associations between perceived achievement pressure and pain, depressive symptoms, and co-occurring pain and depressive symptoms. Although these associations were statistically significant, the RRs were relatively modest in comparison to those elucidated for struggling to cope with pressure. Struggle to cope with pressure was association with pain, while the association was notably higher with depressive symptoms and further stronger with co-occurring symptoms. These results emphasizing that it is not solely the perceived pressure that poses a challenge; rather, it is the surpassing of perceived resources and coping capabilities by perceived pressure that leads to heightened stress perceptions and may contribute to pain and depressive symptoms. Boys who reported struggling to cope with pressure had a higher relative risk for depressive and co-occurring symptoms than girls. One explanation for these differences may be gender differences in coping strategies. Girls often use emotional and social support, while boys may be more likely to use avoidance strategies [33]. Maladaptive coping strategies, such as avoidance, are linked to depressive symptoms in adolescents [34].

The relationship between achievement pressure and pain is in line with previous findings from Wiklund et al. (2012) who found associations between pressure, demands and musculoskeletal pain among Swedish adolescents [35], and a recent Norwegian study which found a significant association between perceived stress and use of over-the-counter analgesics in Norwegian adolescents [36]. Several studies have also identified associations between different types of stress, pressure and depressive symptoms in adolescents [37, 38], but to the best of our knowledge, an association with co-occurring pain and depressive symptoms in adolescents has not been reported previously.

Achievement pressure in this study is related to different areas such as body appearance, academic achievement, sports, and social media. The questions do not specify the sources of this pressure. For instance, whether the pressure comes from the adolescents themselves or from others. Nonetheless, high expectations, whether self-imposed or imposed by others, over time, might give adolescents a feeling of inadequacy, causing stress, which further can contribute to both pain and depressive symptoms. The adolescents’ perception of the pressure and the resources they have to cope with it are fundamental in determining its impact, potentially affecting their health, including pain and depressive symptoms [29]. However, since this is a cross-sectional study, causal mechanisms cannot be determined, as the association might go the other way around. Living with pain and depressive symptoms may for example lead to difficulties in school performance due to high absenteeism, thereby increasing the school pressure [39].

### Implications

The high prevalence of co-occurring pain and depressive symptoms is concerning, particularly as research indicates that children and adolescents facing both conditions exhibit higher levels of functional disabilities compared to those experiencing each condition in isolation [5, 40]. The results highlight the importance of a thorough approach when treating adolescents with either pain, but specifically depressive symptoms, as these symptoms often co-occur. Since girls reported a significantly higher prevalence of all conditions, it is especially important to pay attention to adolescent girls, both clinically and scientifically.

Furthermore, this study draws attention to the relationship between perceived achievement pressure and co-occurring pain and depressive symptoms, as these factors were modestly associated. However, the results indicate that it is not just the feeling of pressure that is important, but rather how well adolescents are able to cope with it. This can potentially be crucial because people working with adolescents can play a vital role in helping young people develop resilience and coping mechanisms to better handle the pressure from today’s society. The significant gender differences in the associations of coping and depressive symptoms and co-occurring symptoms suggests that boys and girls may cope with pressure differently. This highlights the importance of considering gender differences in the development and implementation of interventions aimed at improving coping strategies among adolescents. Further studies should investigate these associations and gender differences in longitudinal designs to better understand the temporal relationships.

### Strengths and limitations

The strength of this study is the large study sample including adolescents from almost all municipalities in Norway, and with the high response rate, enhancing its external validity for Norwegian adolescents. However, boys had more missing data on all the included variables than girls. This differential non-response could have led to an underrepresentation of boys with more symptoms, potentially influencing the prevalence rates and overestimating the gender differences. Another limitation of this study is the use of non-validated questionnaires to measure pressure, coping, and pain. Although these questions have been employed in several studies, they lack formal validation. Additionally, the absence of information on pain severity or the impact of pain and depressive symptoms on adolescent’s daily lives may have overestimated the prevalence and limits the robustness of our measures. Moreover, the cross-sectional nature of this study precludes any inferences about causal mechanisms underlying the identified associations.

## Conclusions

This study revealed a high prevalence of pain and co-occurring pain and depressive symptoms among Norwegian adolescents, with higher prevalence rates observed among girls compared to boys. Perceived achievement pressure was found to be modestly associated with both pain and depressive symptoms and co-occurring pain and depressive symptoms. Moreover, struggling to cope with pressure showed a strong association with all conditions, with a particularly pronounced effect observed in cases of co-occurring problems. Notably, this association was stronger among boys than girls. These findings underscore the importance of addressing adolescents with pain and depressive symptoms from a broad perspective. Enhancing adolescents’ ability to cope with pressure may play a crucial role in the treatment of these conditions.

## Electronic supplementary material

Below is the link to the electronic supplementary material.

**Additional file 1.** Overview of missing variables.

**Additional file 2.** Scores from the subscales of the pressure scale.

## Data Availability

The data that support the findings of this study are available upon reasonable request from the Norwegian Agency for Shared Services in Education and Research (SIKT). Dataset citation required from SIKT: https://doi.org/10.18712/NSD-NSD3007-V3.

## Abbreviations

NOVA: Norwegian Social Research
KORUS: Regional center for drug rehabilitation
STROBE: Strengthening the Reporting of Observational Studies
SIKT: the Norwegian Agency for Shared Services in Education and Research
SES: Socioeconomic status
IQR: Interquartile ranges
RR: Relative Risk Ratio
CI: Confidence Intervals
