# Supplementary material for: Pain and depressive symptoms among adolescents: prevalence and associations with achievement pressure and coping in the Norwegian Ungdata study
Source: BMC Public Health. 2024 Nov 5;24:3054. doi: 10.1186/s12889-024-20566-x (PMC11539564; doi:10.1186/s12889-024-20566-x)
Supplement: Supplementary file 1 — Additional file 1. Overview of missing variables. [file 12889_2024_20566_MOESM1_ESM.docx]

|  | | | |
| --- | --- | --- | --- |
| **Variables** | **Girls n (%)** | **Boys n (%)** | **All n (%)** |
| **School level** | - | - | 0 |
| **Sex** | - | - | 6189 (2.5) |
| **Socioeconomic status** | 777 (0.6) | 1251 (1.0) | 2592 |
| **Pain and depressive symptoms, n (%)** | 10785 (9.7) | 16987 (16.0) | 28893 (11.5) |
| **Perceived pressure, mean (SD)(0-16)** | 6282 (5.4) | 12294 (11.1) | 19365 (8.3) |
| **Coping with pressure, n (%)** | 4606 (3.9) | 10229 (9.0) | 15497 (6.2) |
