# Supplementary material for: Pain and depressive symptoms among adolescents: prevalence and associations with achievement pressure and coping in the Norwegian Ungdata study
Source: BMC Public Health. 2024 Nov 5;24:3054. doi: 10.1186/s12889-024-20566-x (PMC11539564; doi:10.1186/s12889-024-20566-x)
Supplement: Supplementary file 2 — Additional file 2. Scores from the subscales of the pressure scale. [file 12889_2024_20566_MOESM2_ESM.docx]

|  | | | |
| --- | --- | --- | --- |
| **Variables** | **Girls n= 108,071** | **Boys, n= 101,755** | **A4ll, n= 209,826** |
| **Body image pressure n (%)**  No pressure  Little pressure  Some pressure  A lot pressure  Very much pressure | 17,018 (15.7)  31,764 (29.3)  21,495 (19.8)  17,583 (16.2)  20,211 (18.7) | 44,529 (43.7)  33,513 (32.9)  13,400 (13.1)  6,187 (6.0)  4,126 (4.0) | 61,547 (29.3)  65,277 (31.1)  34,895 (16.6)  23,770 (11.3)  24,337 (11.6) |
| **Academic pressure n (%)**  No pressure  Little pressure  Some pressure  A lot pressure  Very much pressure | 8,344 (7.7)  23,166 (21.4)  27,644 (25.5)  26,325 (24.3)  22,592 (20.9) | 21,773 (21.4)  29,735 (29.2)  26,317 (25.8)  15,448 (15.1)  8,482 (8.3) | 30,117 (14.3)  52,901 (25.2)  53,961 (25.7)  41,773 (19.9)  31,074 (14.8) |
| **Sports performance pressure n (%)**  No pressure  Little pressure  Some pressure  A lot pressure  Very much pressure | 32,799 (30.3)  28,715 (26.5)  20,667 (19.1)  14,532 (13.4)  11,358 (10.5) | 44,611 (43.8)  25,494 (25.0)  16,529 (16.2)  9,352 (9.1)  5,769 (5.6) | 77,410 (36.8)  54,209 (25.8)  37,196 (17.7)  23,884 (11.3)  17,127 (8.1) |
| **Social media pressure n (%)**  No pressure  Little pressure  Some pressure  A lot pressure  Very much pressure | 47,439 (43.9)  29,067 (26.9)  15,192 (14.0)  8,665 (8.0)  7,708 (7.1) | 76,564 (75.2)  16,145 (15.8)  5,425 (5.3)  2,006 (1.9)  1,615 (1.5) | 124,003 (59.1)  45,212 (21.5)  20,617 (9.8)  10,671 (5.0)  9,323 (4.4) |
|  | | | |
